# A simple method based on confocal microscopy and thick sections recognizes seven subphases in growth plate chondrocytes

**DOI:** 10.1038/s41598-020-63978-6

**Published:** 2020-04-24

**Authors:** Ángela Fernández-Iglesias, Rocío Fuente, Helena Gil-Peña, Laura Alonso-Duran, María García-Bengoa, Fernando Santos, José M. López

**Affiliations:** 1 Division of Pediatrics, Department of Medicine, Faculty of Medicine, University of Oviedo, CP 33006 Oviedo, Asturias Spain; 2 Fundacion para la Investigación Sanitaria del Principado de Asturias (FINBA), Oviedo, Spain; 3 Department of Morphology and Cellular Biology, Faculty of Medicine, University of Oviedo, Oviedo, Asturias Spain; 4 Pediatric Nephrology, Department of Pediatrics, Hospital Universitario Central de Asturias (HUCA), Oviedo, Asturias Spain

**Keywords:** Cellular imaging, Bone development

## Abstract

This manuscript reports a novel procedure to imaging growth plate chondrocytes by using confocal microscopy. The method is based on fixed undecalcified bone samples, in-block staining with eosin, epoxy resin embedding and grinding to obtain thick sections. It is simple, inexpensive and provides three-dimensional images of entire chondrocytes inside their native lacunae. Quantitative analysis of volume, shape and cytoplasm density of chondrocytes at different strata of the growth plate allowed to objectively grade chondrocytes of the growth plate in seven different clusters. These seven categories of chondrocytes were subsequently evaluated by immunohistochemistry of some well-defined molecular landmarks of chondrocyte differentiation. Furthermore, immunohistochemical analysis of proteins responsible for ionic changes and water transport allowing chondrocyte swelling during hypertrophy was also performed. Results obtained indicate that four subphases can be defined in the pre-hypertrophic zone and three subphases in the hypertrophic zone, a fact that raises that chondrocytes of the growth plate are less homogeneous than usually considered when different zones are defined according to subjective cell morphological criteria. Results in the present study provide a technological innovation and gives new insights into the complexity of the process of chondrocyte differentiation in the growth plate.

## Introduction

Hypertrophy of the chondrocytes of the growth plate is the major contributor to bone lengthening^1^. This process is characterized by a widespread increase of cell volume and has been widely studied, especially with respect to its regulation^2^. However, information regarding how the cell volume increase occurs is relatively scarce. Hypertrophic chondrocytes have great amount of cytoplasm with sparse organelles and numerous membrane transporters, and volume increase is due to unbalanced water accumulation or swelling^3,4^ as well as to synthesis of cytoplasmic components or true hypertrophy^5^. Increase of chondrocyte volume was found to result from three sequential stages by using tomographic phase microscopy^6^: two stages of true hypertrophy (phases 1 and 3) when chondrocytes increase their volume with active synthesis of cytoplasmic components separated by a stage of swelling (phase 2), when volume keeps increasing but synthesis ceases and the cytoplasm density decreases. IGF-I was found to specifically affect chondrocyte hypertrophy during phase 3. These findings supported the view of chondrocyte hypertrophy as a sequence of events, each susceptible of independent regulation, and enabled new possibilities for understanding growth regulation and growth disorders. Unfortunately, no further studies in this line of work have been published. A possible cause is that diffraction phase microscopy is not commercially accessible and is not available in most laboratories. Thus, we have developed a procedure based on confocal microscopy that allows the analysis of cell volume, cell shape and cytoplasm density during chondrocyte maturation. This approach is simple, inexpensive, provides three-dimensional images of chondrocytes that maintain their shape and size inside their native lacunae and enables objective morphological discrimination of discrete groups of chondrocyte populations in the growth plate cartilage. For further molecular characterization, the expression profiles of well-defined molecular landmarks of distinct stages of chondrocyte differentiation were immunohistochemically analyzed with respect to populations of chondrocytes graded by the quantitative analysis. Our study corroborates that chondrocyte hypertrophy is a multistage process and reports for the first time that four subphases can be defined in the pre-hypertrophic zone and three subphases in the hypertrophic zone.

## Materials and Methods

### Animals

The study was carried out in six weeks old female Sprague-Dawley rats (Charles River Laboratories, L´Arbresle, France). Procedures involving animals and their care were conducted according to Spanish law on the use of experimental animals, which acknowledges the European Directive 86/609. The project proposal was approved by the Ethical Committee of University of Oviedo, Spain. Rats were housed in individual cages under controlled conditions of light (12 light/dark cycles) and temperature (21–23 °C) with free access to rats’ standard diet and tap water. Animals were sacrificed under lethal dose of Dolethal® anaesthesia.

This work comprised three consecutive steps. First, we established a procedure for processing the growth plate which would result in the preservation of the structure of the hypertrophic chondrocytes and be suitable to obtain three-dimensional images of whole (non-sectioned) chondrocytes inside their native lacunae by using confocal microscopy. A group of twelve rats was used for this first study. The second step was to apply the procedure for analysis of cell changes during chondrocyte proliferation and hypertrophy in the growth plate. A cluster analysis was performed to classify chondrocytes according to quantitative analysis of volume, shape and cytoplasm density. Finally, an immunohistochemical study of the expression of proteins associated with the process of chondrocyte differentiation in the growth plate cartilage was performed in order to further characterize the different classes of subpopulations discriminated by their morphological features.

### Procedure

Undecalcified bones were fixed under osmotically controlled conditions, *in block* stained, embedded with epoxy resin and thinned by grinding to obtain 100-μm thick bone sections. Different techniques for fixation, staining and post-processing were tested for their effectiveness and were systematically optimized for best synergy. Detailed information about the studies, including bone sampling, fixative solutions, dye concentrations and pH, staining times, embedding and ground section preparation is presented in Supplementary Information.

Thick bone sections were imaged with a confocal microscope Leica TCS SP8 (Leica Microsystems, Germany) equipped with a pulsed white light laser (470–670 nm), Acousto-Optical Beam Splitter (AOBS) and two internal hybrid single photon counting detectors and operated by Leica Application Suite X program (Leica Microsystems, Wetzlar, Germany). Excitation and emission lambda scans were obtained by scanning the excitation (absorbance) spectrum of the sample while simultaneously acquiring the fluorescence emission spectrum at each excitation wavelength coordinate. Excitation-emission maps were obtained for positive samples resulting from different fixative and staining combinations and for controls samples for native and induced autofluorescence. The image acquisition settings for negative controls were designed to maximize the ratio of fluorescence over autofluorescence. Autofluorescence-corrected images were obtained by digital subtraction of the autofluorescence from the fluorescence of positive samples. Ultrastructural images of the cytoplasm of chondrocytes were alto obtained by transmission electron microcopy and these images were used as a reference for judging the quality of the micro-structural data in images obtained from confocal microscopy. Tibial samples for electron microscopy were fixed in a solution of 2.5% glutaraldehyde and 0.7% RHT (Strem Chemicals, Newburyport, MA) in 0.05 M cacodylate buffer, pH 7.4, for 3 hours at 4 °C. Then, the samples were washed in buffer and postfixed in a solution of 1% osmium tetroxide and 0.7% RHT in cacodylate buffer for 2 hours at room temperature, dehydrated with a graded series of acetone and embedded in Durkupan-ACM (Sigma). Ultrathin sections were cut on a Reicher Ultracut E ultramicrotome, stained with lead citrate and viewed with a Jeol JEM-2000 EX II electron microscope.

Samples for confocal microscopy were first scanned at low magnification (20×) to locate growth plate cartilage. Confocal slide scanning was then performed using a 63X oil immersion objective with 1.4 NA at two different areas of hypertrophic cartilage. The pixel intensity, ranging from 0 to 255, was set to be the mean value of four scans. The increment of the Z-axis optical section was 0.5 μm to obtain 100 continuous images and those images were sequentially overlapped along the z-axis to form a stack of 184.52 μm (x) × 184.52 μm (y) × 50 μm (z) with X/Y resolution of 1024×1024 pixels. Twenty chondrocytes, all situated in the last three rows of the hypertrophic cartilage adjacent to the invading front, were analyzed in each sample. A trained operator, the same for all samples, used a semi-automated, hand-drawn contouring system to delineate each chondrocyte. Structural parameters, including cell volume, sphericity and ellipticity were obtained by using the SURPASS software. The Leica LAS X 3D software and the IMARIS v.7.1.1. software (Bitplane, Switzerland) image reconstruction software were used for the 3D projection and analysis of the confocal micrographs. In Supplementary Videos 1 and 2, the image stacks were displayed using the “Ortho Slice” function, and the video was made via the “Movie Maker” function with the increase in display time in association with the depth of the optical section. The quality of the preservation was evaluated in each sample by scoring 20 chondrocytes located in the last two rows of the hypertrophic chondrocyte layer. Three issues were considered: structural integrity (SI), cytoplasm preservation (CP) and integrated optical density (IOD). SI was considered optimal when hypertrophic chondrocytes were regularly attached to the pericellular matrix border so that they completely fill their lacunae and neither irregular shrinkage nor cell collapse or lysing were observed. It was rated by using a semiquantitative scale ranging from 1–4 (1 poor; 2 fair; 3 good; and 4 excellent). CP was considered optimal when the cells were not vacuolated and had a homogeneous content and semiqualitative evaluation was performed in the same way (1 poor; 2 fair; 3 good; and 4 excellent). IOD was considered as an indicator of cytoplasmic density and was calculated by quantifying the fluorescent signal. The entire volume of the chondrocytes was measured, and every pixel was used to calculate IOD from which was subtracted the fluorescence of the background control. Values of IOD obtained for all chondrocytes of the different were ranged in descending order and rated from 1–4 (4 for the top 25% of the distribution; 3 for those between 25% and 50%; 2 for those between 50% and 75%; and 1 for those between 75% and the bottom of the distribution). The cumulative score was calculated as the sum of SI, CP and IOD (range 3–12) to determine the final score (FS) value. Values obtained from the chondrocytes in each group (n = 20) were expressed as X ± SD and the group with the highest X and lowest SD was finally selected.

### Analysis of cell changes during chondrocyte hypertrophy

Once the procedure was established, it was applied to a group of five rats to analyze changes in cell volume, cell shape and cytoplasm density during chondrocyte maturation. To this end, both tibias of each rat were processed as previously described to obtain a total of 20 positive and 20 autofluorescence-control samples that were analyzed with confocal microscopy. Four complete columns of chondrocytes, extending from proliferating to the hypertrophic zones, were scanned in each sample using the same objective (63×) and image acquisition settings previously reported. Two or three scans were performed along the XY plane, sequential in the Y axis, to obtain an image reconstruction of a complete column and the sequence of cell structural variation along a vertical column, by measuring a total of 500 chondrocytes, was analyzed. In Supplementary Videos 3 and 4.

### Immunohistochemistry

In order to connect volume, shape and cytoplasm density changes in specific chondrocyte subsets with chondrocyte progression through proliferation and hypertrophy we performed immunohistochemistry of some well-defined molecular landmarks of distinct stages of chondrocyte differentiation. We analyzed the immunolocalization of collagen type II (Col2a1), collagen type X (Col10a), transcription factor Sox9, insulin like growth factor 1 (Igf1), aquaporin 1 (Aqp1) and Na-K-Cl cotransporter 1 (NKCC1) with respect to the seven clusters of chondrocytes objectively graded by the quantitative analysis. To this end, an additional group of five rats was used. Tibias were dissected, fixed in 4% paraformaldehyde and embedded in methyl-methacrylate, as previously described by our group^7^. Immunodetection was carried out on 5-μm-thick methyl methacrylate sections as further described in Supplementary methods.

### Statistical analyses

To investigate differences in the quality of the preservation and labelling of hypertrophic chondrocytes, X and SD of the final rating were obtained for each of the 24 groups resulting from combinations of fixative and staining solutions. Comparison among groups was performed using the one-way ANOVA following by a Turkey’s Multiple Comparison test. All statistical analyses utilized a 95% confidence level and were conducted using GraphPad Prism statistical software v.7 (La Jolla, California, USA).

A cluster analysis was used for classifying chondrocytes according to the values obtained for the cell parameters on the basis of the distance between them in a multidimensional array. A hierarchical dendrogram showing the order of successive agglomerations was generated and the cluster number was chosen by applying the Ward’s linkage algorithm in combination with Manhattan distances. The statistical significance among clusters was assessed by one-way ANOVA followed by a Turkey’s Multiple Comparison test.

## Results and Discussion

### Processing procedure

Analysis of bone samples at the spectral confocal microscope showed that both the quality of the preservation and the IOD of hypertrophic chondrocytes varied noticeably depending on fixation and staining conditions (Table 1). The FS varied between 6.4 and 9.9 (12 being the theoretical maximum). Two procedures reached the highest value (9.9): (1) fixation in glutaraldehyde 2%, ruthenium hexaammine trichloride 0.5% and calcium chloride 5 mM in 0.025 M sodium cacodylate buffer (pH 7.4, osmolarity 300 mOsm) (F3) followed by staining with a solution of eosin 0.5% in acetate buffered ethanol 60% pH 4.8 during 2 hours at 4 °C and (2) the same fixation followed by staining with a solution of eosin of higher concentration (1%) at the same pH (4.8) for a longer time (4 hours). When values for the three simple parameters in the two procedures were compared it was found that both SI and CP were higher in the first procedure (3.40 vs 3.25 and 3.60 vs 3.45, respectively) whereas the second procedure showed a higher value for IOD (3.2 vs 2.9). The quality of cell preservation was prioritized over fluorescence intensity and the procedure using lower eosin concentration during less time was selected. Although the penetrating properties of formaldehyde are stronger than those of glutaraldehyde, this distributed homogeneously into the slices of about 2 mm in thickness of the growth plate and effectively preserved the structure of hypertrophic chondrocytes giving good overall cytoplasmic and nuclear detail. Formaldehyde, limited to low concentrations because of its high osmolarity, did not improve structural preservation, slightly increased cell shrinkage and considerably decreased signal-to-background fluorescence ratios. Ruthenium hexammine trichloride was effective to prevent the loss of matrix proteoglycans and shrinkage of chondrocytes at the two concentrations tested (0.5 and 0.7%) and thus, the 0.5% concentration was chosen. Addition of calcium chloride to the fixative improved structural preservation of chondrocytes while not decreasing signal-to-background autofluorescence ratio. These results confirm that minor changes during tissue processing induce noticeable changes in the preservation of hypertrophic chondrocytes^8^.

**Table 1:** Effect of different fixation/staining protocols on the preservation quality and staining of hypertrophic chondrocytes.

		Parameters	STAIN							
			Eosin 0.5%				Eosin 1%			
			pH 4.8		pH 5.5		pH 4.8		pH 5.5	
			2 h	4 h	2 h	4 h	2 h	4 h	2 h	4 h
**FIXATIVE**	**F1**	**SI**	2.45 ± 0.51	2.50 ± 0.51	2.55 ± 0.51	2.45 ± 0.51	2.30 ± 0.47	1.90 ± 0.55	2.15 ± 0.67	2.00 ± 0.46
		**CP**	2.65 ± 0.49	2.75 ± 0.44	2.75 ± 0.44	2.65 ± 0.49	2.75 ± 0.55	2.25 ± 0.55	2.40 ± 0.60	2.25 ± 0.44
		**IOD**	2.45 ± 1.28	2.70 ± 1.13	1.70 ± 0.80	2.00 ± 1.03	2.75 ± 1.21	2.95 ± 1.10	1.85 ± 0.88	2.20 ± 1.06
		**FS**	7.55 ± 1.70	7.95 ± 1.43	7.00 ± 1.26	7.10 ± 1.55	7.80 ± 1.54	7.10 ± 1.29	6.40 ± 1.31	6.45 ± 1.23
	**F2**	**SI**	3.30 ± 0.47	3.15 ± 0.59	2.80 ± 0.52	2.65 ± 0.49	3.00 ± 0.46	2.95 ± 0.51	2.70 ± 0.47	2.40 ± 0.68
		**CP**	3.40 ± 0.50	3.35 ± 0.49	3.10 ± 0.45	2.85 ± 0.37	3.10 ± 0.31	3.00 ± 0.46	3.00 ± 0.56	2.55 ± 0.51
		**IOD**	2.50 ± 1.36	2.75 ± 1.16	1.85 ± 0.93	2.65 ± 1.18	3.00 ± 1.03	3.15 ± 0.93	2.05 ± 0.83	2.30 ± 1.08
		**FS**	9.20 ± 1.64	9.25 ± 1.37	7.75 ± 1.37	8.15 ± 1.39	9.10 ± 0.97	9.10 ± 1.25	7.75 ± 1.33	7.25 ± 1.41
	**F3**	**SI**	3.40 ± 0.60	3.30 ± 0.57	3.45 ± 0.51	3.10 ± 0.64	3.20 ± 0.62	3.25 ± 0.64	3.15 ± 0.59	3.20 ± 0.70
		**CP**	3.60 ± 0.50	3.55 ± 0.51	3.30 ± 0.47	3.20 ± 0.52	3.40 ± 0.50	3.45 ± 0.51	3.30 ± 0.47	3.35 ± 0.59
		**IOD**	2.90 ± 1.12	2.95 ± 1.00	1.95 ± 0.89	2.65 ± 1.14	3.15 ± 0.93	3.20 ± 0.89	2.10 ± 0.97	2.30 ± 1.03
		**FS**	9.90 ± 1.21	9.80 ± 1.47	8.70 ± 1.22	8.95 ± 1.67	9.75 ± 1.07	9.90 ± 1.37	8.55 ± 1.15	8.85 ± 1.31

Three fixative solutions (F1, PFA 0.5%, Glu 1.2% and RHT 0.5% in 0.025 M SCB; F2, Glu 2.5% and RHT 0.7% in 0.03 M SCB; and F3, Glu 2%, RHT 0.5% and CaCl_2_ 5 mM in 0.025 M sodium cacodylate buffer), two eosin concentrations (0.5% and 1%) at two different pH (4.8 and 5.5) and two staining times (2 and 4 h), in a total of 24 groups. PFA, paraformaldehyde; Glu, glutaraldehyde; RHT, ruthenium hexaammine trichloride; SCB, sodium cacodylate buffer; CaCl_2_, calcium chloride. SI, structural integrity; CP, cytoplasm preservation; IOD, integrated optical density; FS, final score, (see text for details). Each point is the mean value of 20 hypertrophic chondrocytes ± SD

**Figure 1:**
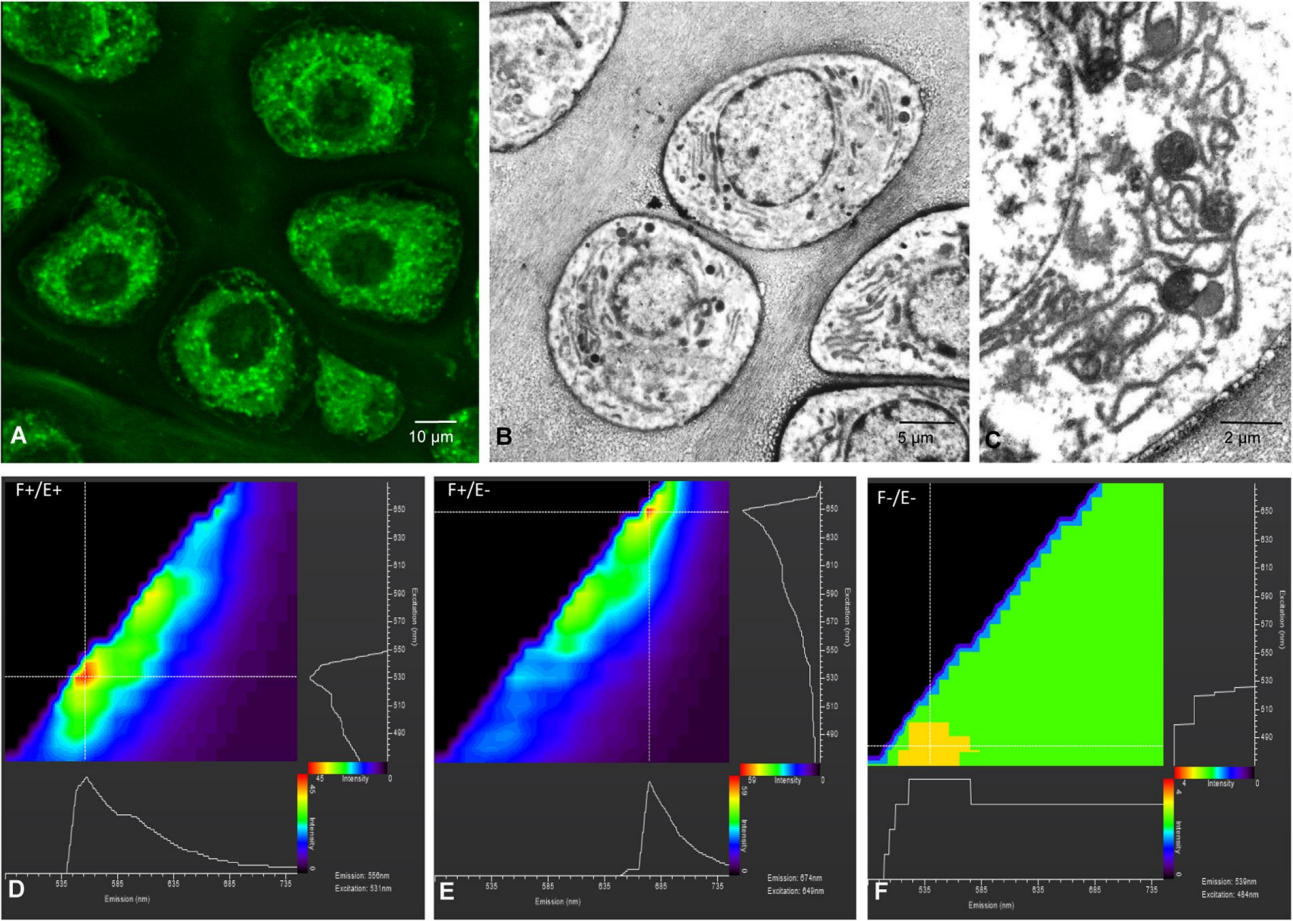
(**A**) Confocal micrograph of a single optical section 10 µm from the surface of the block showing prehypertrophic chondrocytes with fluorescent labelling in form of a reticular network around the nucleus. (**B**,**C**) are transmission electron micrographs of prehypertrophic chondrocytes with rough endoplasmic reticulum, scattered mitochondria and some lipid droplets. A correspondence is found between the fluorescent labelling and the Intracellular organelles viewed with the electron microscopy. (**D**–**F**) are excitation-emission maps of a positive sample (F+/E+) and two control samples for background fluorescence: F+/E−, fixed non-eosin stained; F−/E−, non-fixed and non-eosin stained. The map of F+/E+ shows a broad range of effective excitation wavelengths and peaks in intensity at excitation/emission wavelengths of 531/556 nm. The map of F+/E− shows a significant decrease of fluorescence and the excitation/emission peak is found at 649/674 nm. Native autofluorescence due to the intrinsic components of the chondrocytes (F−/E−) was low. Color scale bar represents fluorescence intensity in arbitrary units.

Chondrocytes showed a strong and clean fluorescence signal dispersed throughout the cytoplasm (Fig. 1A). The fluorescence signal appeared diffused in the intercellular matrix and almost absent in the nuclear region. Fluorescence signal was more intense in the perinuclear region and appeared constituted by a network of granulo-filamentous structures. Comparative analysis of confocal and electron micrographs (Fig. 1A–C) revealed that fluorescent signal correspond to the area where organelles were concentrated. Perinuclear cytoplasm contained abundant rough endoplasmic reticulum, scattered mitochondria and some lipid droplets. The excitation-emission map of the samples treated with the selected procedure (Fig. 1D–F) showed a broad range of effective excitation wavelengths and peaks in intensity at excitation/emission wavelengths of 531/556 nm. Matched control samples fixed in the same way but without eosin staining showed a clear different map in which the peak at 531/556 nm disappeared and a smaller one was found at 649/674, a result that demonstrates that a substantial fraction of the fluorescence signal in the first sample came from eosin labelling.

**Figure 2:**
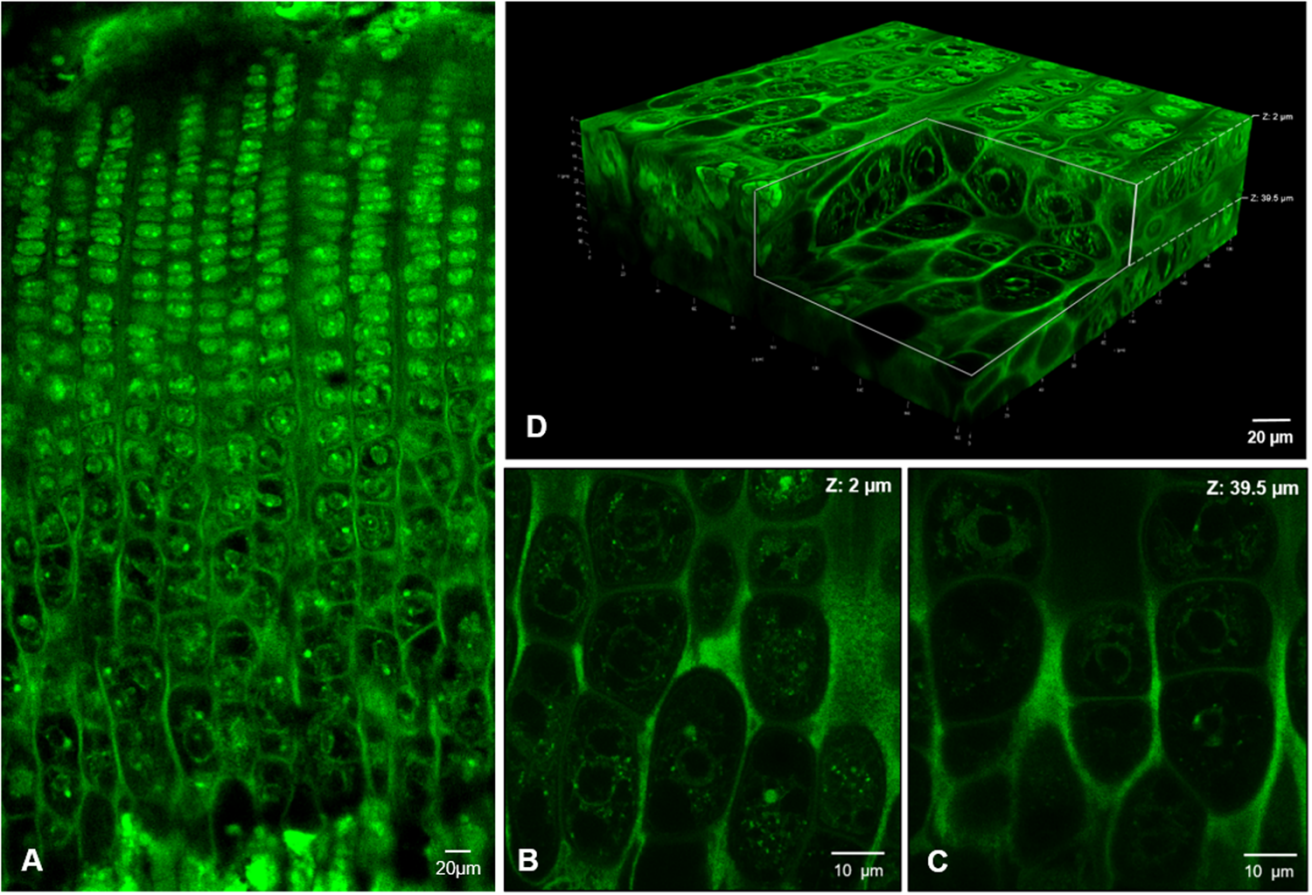
Confocal imaging of the growth plate cartilage. (**A**) Low-power micrograph showing the characteristic arrangement of the chondrocytes stacked in columns and the gradual variation in size, shape and cytoplasm density. (**B**,**C**) are two optical sections of the same field at different focal planes (2 and 39.5 µm, respectively). 3D projection of the thick section of the growth plate columns. Three-dimensional visualization is shown in Supplementary Videos.

Fluorescent labelling was observed in chondrocytes throughout the growth plate, with highest levels in proliferative chondrocytes and a progressively decreasing levels towards the hypertrophic zone (Fig. 2). Analysis of thick sections with the confocal microscopy avoids damage to the integrity of the chondrocytes caused by mechanical sectioning and this allows to visualize entire chondrocytes inside their native lacunae preserving cell-matrix interactions. Eosin has quite good penetration into thick tissue sections of growth plate cartilage and stains the cytoplasm of the chondrocytes providing a strong fluorescent signal that resisted solvent dehydration, epoxy resin embedding and heat polymerization. Eosin is usually not regarded as a fluorochrome but results in the present work shows its utility for fluorescence labelling of chondrocytes, and this agrees with some previous studies in which eosin staining and confocal microscopy were used to analyze the three-dimensional distribution of cytoplasmic constituents of protein nature^9–11^ and also for tissue pathology diagnosis^12,13^. Although eosin possesses only 20% the fluorescence quantum yield of more conventional fluorophores, such as fluorescein and rhodamine, eosin conjugated to either an immunoglobulin G (IgG) or steptavidin has been also proposed as an alternative to fluorescein in immunofluorescence^14,15^. Furthermore, eosin forms protein–dye complexes whose absorbance is proportional to the concentration of protein^16–18^ and it has been used as a fluorescent probe for estimating a wide range of proteins by spectroscopy^19–21^.

It is also of note that embedding of the growth plate in epoxy resin reduced refractive disparity between the different tissue constituents and this decreased random light scattering and increased penetration of light for imaging. Analysis of the maximum depth at which images can be obtained in the samples revealed that there was effective photon penetration along the Z-axis up to 70 microns depth for both the excitation laser and the emission fluorescence, being the detector gain was adjusted from 36% (surface) to 57% (Z: 70 μm). As a result, the three-dimensional reconstruction of focal image stacks of hypertrophic chondrocytes allowed to obtain images of intact entire chondrocytes inside their lacunae with a relatively smooth surface and minimal irregular shrinkage or cell collapse even for those adjacent to the vascular invasion front. The complete serial optical sections, from the surface to depth = 70 μm, are shown in Supplementary video 1.

The findings obtained in this study support the usefulness of this easy and inexpensive approach based on *in block* eosin staining followed by embedding in epoxy resin and grinding to analyze growth plate chondrocytes by confocal microscopy.

### Analysis of cell changes during chondrocyte proliferation and hypertrophy

**Figure 3:**
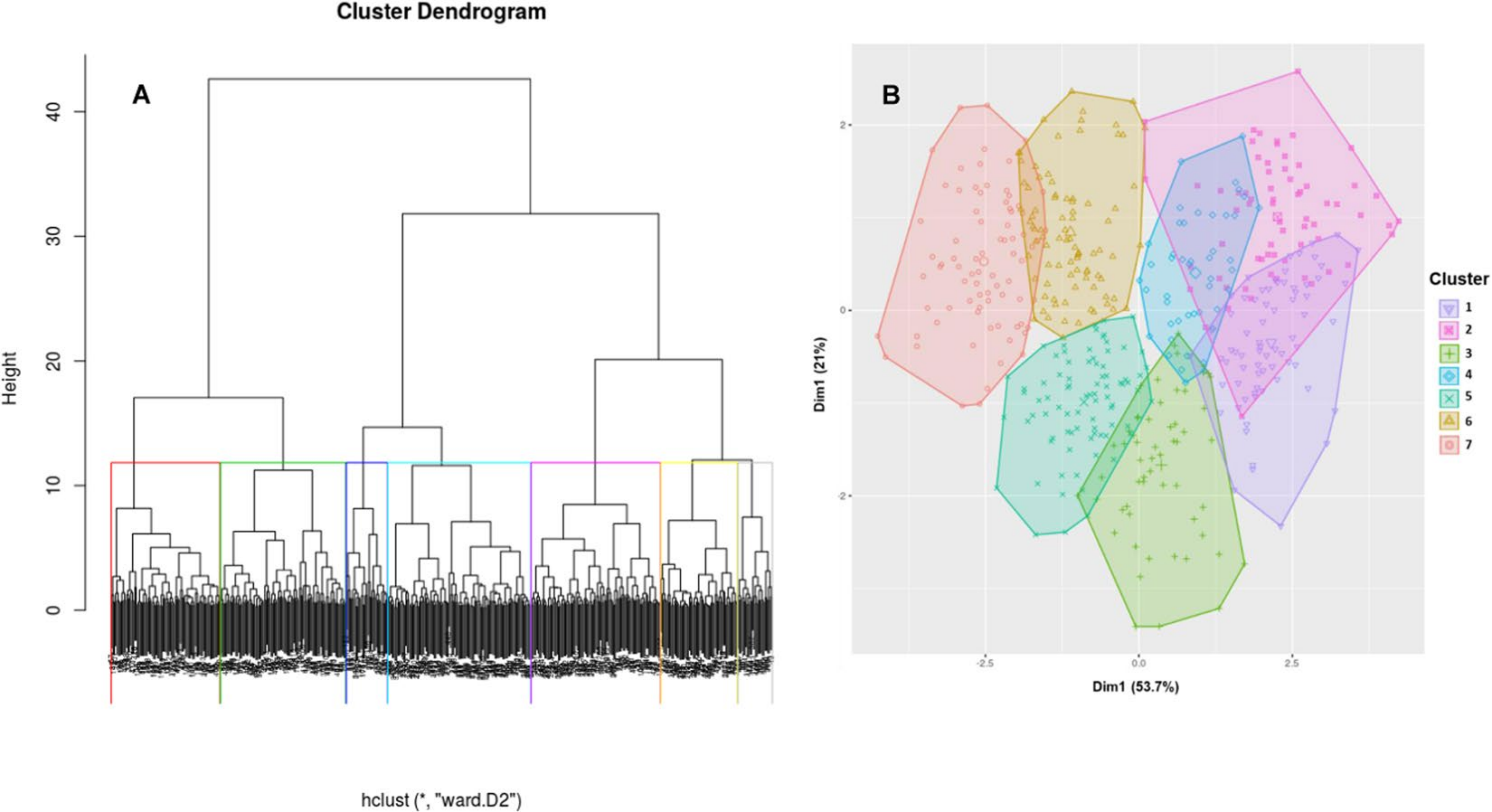
Representation of the cluster analysis. (**A**) Dendrogram showing the order in which chondrocytes were clustered by applying the Ward’s linkage algorithm in combination with Manhattan distances. (**B**) Two-dimensional representation of the cluster solution. Chondrocytes are represented by points in the plot, using principal parameters and an ellipse was drawn around each cluster.

**Figure 4:**
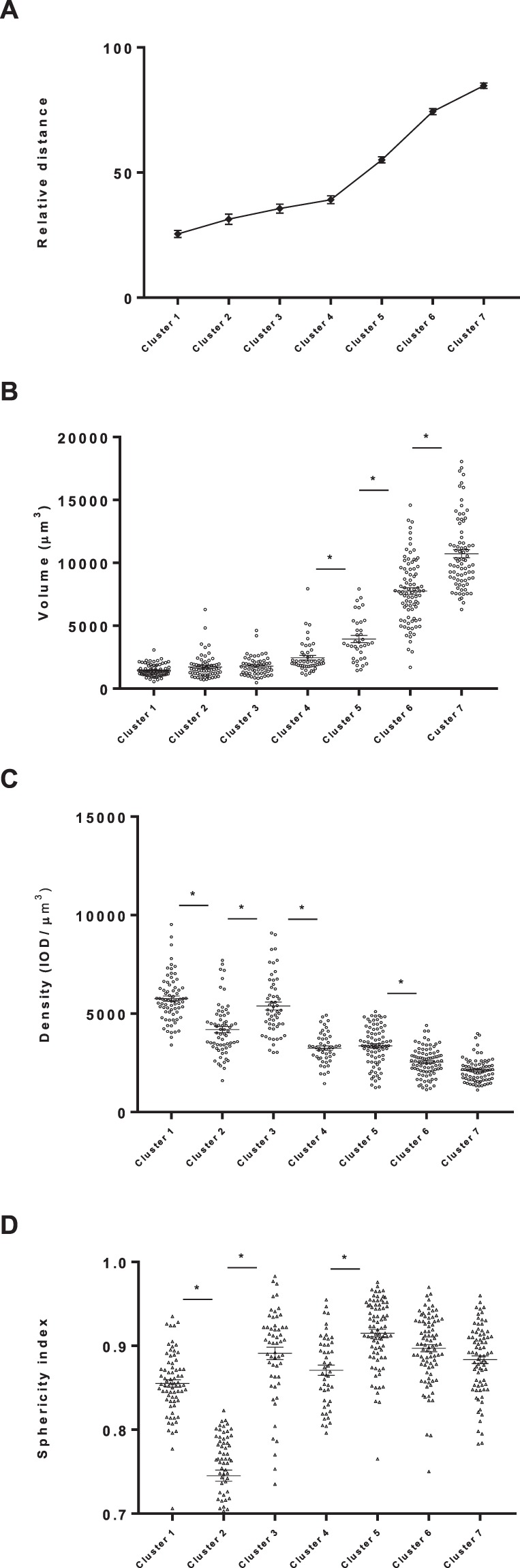
Quantitative values of relative distance, volume, cytoplasm density and sphericity index of chondrocytes in the seven categories that resulted for the cluster analysis. Data obtained from a total of 500 chondrocytes measured. Asterisks indicate significance (P < 0.05) when compared to preceding cluster.

Size, shape and cytoplasm density of the chondrocytes disposed in vertical columns exhibit a range of continuous variation from the top to the bottom. Growth plate shows a multilayered horizontal stratification but precise transition lines between contiguous zones cannot be established because variation is continuous. Quantitative values of cell volume, shape and cytoplasmic density obtained from each chondrocyte were used to perform a hierarchical cluster analysis and to objectively grade the chondrocytes of the growth plate. Comparison in a multidimensional array of quantitative values allowed to define distances between chondrocytes and to establish seven categories or clusters. Chondrocytes in a particular cluster share common characteristics and are more similar to each other than to those in the other clusters. A hierarchical dendrogram showing the order of successive agglomerations and a cluster plot are shown in Fig. 3. Four layers corresponding to different stages of differentiation are classically considered in the growth plate: resting, proliferating, prehypertrophic, and hypertrophic. Results obtained in the present analysis show some of these layers defined by subjective cell morphological criteria can be subdivided into several subphases. According to our results, chondrocyte cell volume remains unchanged in the clusters 1 to 4 and then a statistically significant increase takes place in the transition from cluster 4 to cluster 5 (Fig. 4). Since cell enlargement triggers the switch to hypertrophy, it could be considered that transition from cluster 4 to cluster 5 corresponds to the transition from the proliferating to the hypertrophic stage. Thus, cluster analysis allows to differentiate four subphases in the pre-hypertrophic stage (clusters 1 to 4) and three subphases in the hypertrophic stage (clusters 5 to 7). When clusters of the hypertrophic stage were analyzed it was found that transition from cluster 4 to cluster 5 was associated with a significant increase in cell volume without significant change in cytoplasm density. However, an increase of cell volume and decrease of the cytoplasmic density, both statistically significant, took place in the transition from cluster 5 to cluster 6. Finally, cell volume increase without cytoplasmic density variation occurred in the transition from cluster 6 to cluster 7.

**Figure 5:**
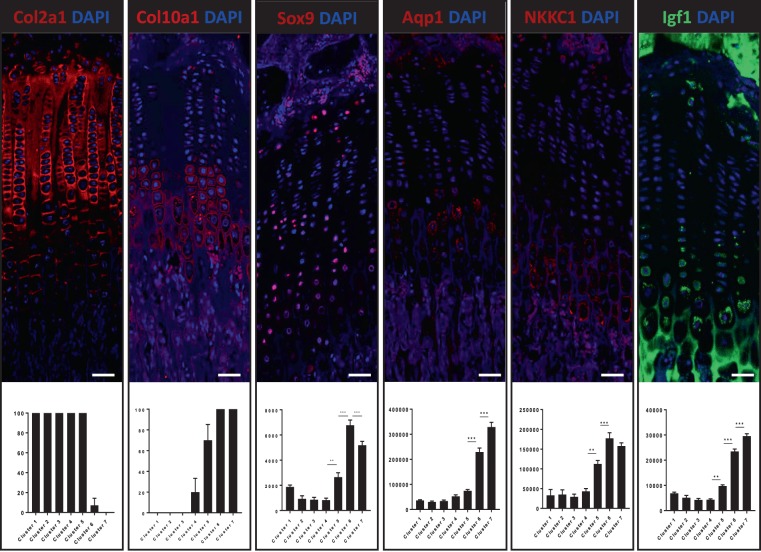
Immunohistochemical analysis of the expression of Col2a1, Col10a1, Sox9, Aqp1, NKCC1 and Igf1 in the seven categories that resulted for the cluster analysis. Asterisks indicate significance (P < 0.05) when compared to preceding cluster.

The seven categories of chondrocytes were evaluated by immunohistochemistry of chondrocyte markers of maturity and activity, including Col2a1, a structural component of the cartilage matrix that may act as an extracellular signaling molecule suppressing chondrocyte hypertrophy; Col10a1, a short-chain collagen associated with hypertrophy of chondrocytes and involved in the ossification of cartilage; Sox9, a transcription factor essential for chondrocyte differentiation; Igf1, a growth factor that stimulates both proliferation and hypertrophy of chondrocytes by paracrine/autocrine mechanisms; Aqp1, a water channel protein that is responsible for high water permeability of chondrocyte membrane during hypertrophy; and NKCC1, a secondary active transport system that is responsible for cell volume regulation. Immunohistochemical analysis of growth plate cartilage (Fig. 5) showed that Col2a1 is expressed throughout the growth plate but it decreases as chondrocytes become hypertrophic and ceases in the chondrocytes near the zone of mineralization. Analysis of immunohistochemical staining demonstrated intense signal in clusters 1 to 5 but it significantly decreased in the transition from cluster 5 to cluster 6 whereas no signal was observed in the cluster 7. By contrast, Col10a1 had the inverse distribution of Col2a1, signal was absent in clusters 1 to 3, raised in cluster 4, increased in cluster 5 and reached high levels in clusters 6 and 7. The restricted expression of Col10a1within the hypertrophic zone of the growth plate supports that it enables the removal of type II collagen fibrils and participates in the mineralization process. Sox 9 was observed throughout the growth plate in all chondrocyte layers, with low values in clusters 1 to 4 and significant increases in the transitions from cluster 4 to cluster 5 and from cluster 5 to cluster 6 where it peaks. Finally, it significantly decreased in the transitions from cluster 6 to cluster 7. These results are coincident with previous reporting that Sox9 has essential roles in successive steps of the chondrocyte differentiation pathway^22,23^. Aqp1 and NKCC1 proteins showed equally low signal levels in clusters 1 to 4 but NKCC1 exhibited a significant increase in the transition from cluster 4 to cluster 5 whereas Aqp1 expression remained low in the cluster 5. Both transport proteins showed high signal levels in cluster 6, where NKCC1 reached the peak level to slightly decrease in cluster 7, although without significant difference. However, Aqp1 signal continuously increased during hypertrophy and reached the highest level in cluster 7. Such differences in the expression profiles during chondrocyte hypertrophy suggest that NKCC1 may have a major role in the onset of cell volume expansion (cluster 5) whereas Aqp1 may have its main function in the later stages of the process (cluster 7). One major factor that determines ion mobility and the net movement of water into the chondrocyte is the interaction with an ionic microenvironment with high cationic content due to the concentration of fixed negative charges on proteoglycans of the cartilage extracellular matrix. Interactions between transported molecules with the microenvironment and both active and passive membrane transport proteins have been documented in cell volume regulation of chondrocytes^4,5^. Igf1 expression was in the clusters 1 to 4 and a significant increase occurred in the transition from cluster 4 to cluster 5. Likewise, significant increases were also found in the transition from cluster 5 to cluster 6 and in the transition from cluster 6 to cluster 7, where reached the highest expression level. This data supports the view that Igf1 promotes the anabolic actions associated with the increased biosynthetic activity of chondrocyte hypertrophy^24^.

The occurrence of four clusters in the pre-hypertrophic stage is remarkable because only two categories of chondrocytes, resting and proliferating, have so far been defined. This result implies that proliferating zone has a less uniform structure than usually considered. Previous works have reported that chondrocytes possess nearly the same structure from the beginning to the end of the proliferating zone^25^. However functional differences among the various segments of the columns have also been reported. The establishment of clonal columns of chondrocytes is a major process that precisely occurs at the transition from the resting to the proliferating zone. Likewise, the rate of cell proliferation varies through the proliferating zone of the growth plate and changes in gene expression associated with regional differences in proliferative activity have been reported^26,27^. Since cells approximately double in size prior to mitosis and this involves both protein synthesis and energy production, it is expected that different proliferation activity may result in structural differences. Immunohistochemical analysis revealed that no differences among clusters 1 to 3 are found for Col2a1, Col10a1 or Sox9. Type II collagen and Sox9 remained unchanged at cluster 4 but Col10a1expression was first found at some chondrocytes of this cluster. Since chondrocyte cell volume remained unchanged in the clusters 1 to 4 and significantly increased in the transition from cluster 4 to cluster 5, it could be concluded that Col10a1expression is turned on before the beginning of cell volume increment. Results obtained in the present work suggest that quantitative analysis of volume, shape and cytoplasm of chondrocytes could be a specific and sensitive method to detect differences in the activity of proliferative chondrocytes.

Our results obtained on chemically-fixed *in situ* chondrocytes are basically coincident with those obtained by Cooper and colleagues on living chondrocytes dissociated from the growth plate^6^. Fixed cells conserve their three-dimensional interaction with the surrounding extracellular matrix and preserve their shape and size but lose some of their low-molecular-weight intracellular constituents and lipids. Living chondrocytes dissociated from the growth plate maintain life functions but lose their specific shape and acquire spherical shape. The fact that two approaches with different advantages and disadvantages lead to the same conclusion reinforces the assumption that chondrocyte volume enlargement during hypertrophy is a multistage process consisting of three phases. Furthermore, our results show that NKCC1 expression is involved in the beginning of the increment in cell volume and the expression profiles of NKCC1 and Aqp1 specifically changed during different phases of chondrocyte hypertrophy.

In summary, the new method reported in the present work allows obtaining additional information to common measurements of chondrocytes and improves the understanding of the sequence of events during the process of differentiation of these cells. This is remarkable because chondrocytes go through a series of orderly changes where each stage depends on successful completion of the stage before and the correct sequence of changes is of major importance for the proper formation of the bones.

## Supplementary information

Supplementary Video 1.

Supplementary Video 2.

Supplementary Video 3.

Supplementary Video 4.

Supplementary Methods.

## References

[1] Hall, B. K. *Bones and Cartilage: Developmental and Evolutionary Skeletal Biology* (2015).

[2] Kronenberg H (2003). Developmental regulation of the growth plate. Nature.

[3] Bush PG, Pritchard M, Loqman MY, Damron TA, Hall AC (2010). A key role for membrane transporter NKCC1 in mediating chondrocyte volume increase in the mammalian growth plate. J. Bone Miner. Res..

[4] Loqman MY, Bush PG, Farquharson C, Hall AC (2013). Suppression of mammalian bone growth by membrane transport inhibitors. J. Cell. Biochem..

[5] Bush PG, Parisinos CA, Hall AC (2008). The osmotic sensitivity of rat growth plate chondrocytes *in situ*; clarifying the mechanisms of hypertrophy. J. Cell. Physiol..

[6] Cooper KL (2013). Multiple phases of chondrocyte enlargement underlie differences in skeletal proportions. Nature.

[7] Fuente R (2018). Marked alterations in the structure, dynamics and maturation of growth plate likely explain growth retardation and bone deformities of young Hyp mice. Bone.

[8] Loqman MY, Bush PG, Farquharson C, Hall AC (2010). A cell shrinkage artefact in growth plate chondrocytes with common fixative solutions: importance of fixative osmolarity for maintaining morphology. Eur. Cell. Mater..

[9] de Carvalho HF, Taboga SR (1996). Fluorescence and confocal laser scanning microscopy imaging of elastic fibers in hematoxylin-eosin stained sections. Histochem. Cell Biol..

[10] Wu Y, Li B, Gao XM (2002). Selective fluorescence of zymogen granules of pancreatic acinar cells stained with hematoxylin and eosin. Biotech. Histochem..

[11] Capani F (2001). Phalloidin-eosin followed by photo-oxidation: a novel method for localizing F-actin at the light and electron microscopic levels. J. Histochem. Cytochem..

[12] Luo T, Lu Y, Liu S, Lin D, Qu J (2017). Enhanced Visualization of Hematoxylin and Eosin Stained Pathological Characteristics by Phasor Approach. Anal. Chem..

[13] Castellanos MR, Nehru VM, Pirog EC, Optiz L (2018). Fluorescence microscopy of H&E stained cervical biopsies to assist the diagnosis and grading of CIN. Pathol. Res. Pract..

[14] Hulspas, R., Bioconsulting, C. T. & Keij, J. F. Avidin-EITC: an alternative to avidin-FITC in confocal scanning laser microscopy. *J. Histochem. Cytochem*., 10.1177/41.8.7687265 (1993). 10.1177/41.8.7687265 7687265

[15] Deerinck TJ (1994). Fluorescence photooxidation with eosin: a method for high resolution immunolocalization and *in situ* hybridization detection for light and electron microscopy. J. Cell Biol..

[16] Waheed AA, Rao KS, Gupta PD (2000). Mechanism of dye binding in the protein assay using eosin dyes. Anal. Biochem..

[17] Waheed AA, Gupta PD (1996). Application of an eosin B dye method for estimating a wide range of proteins. J. Biochem. Biophys. Methods.

[18] Jordanides XJ, Lang MJ, Song X, Fleming GR (1999). Solvation Dynamics in Protein Environments Studied by Photon Echo Spectroscopy. J. Phys. Chem. B.

[19] Waheed AA, Gupta PD (2000). Estimation of submicrogram quantities of protein using the dye eosin Y. J. Biochem. Biophys. Methods.

[20] Ni Y, Liu Q, Kokot S (2011). Spectrophotometric study of the interaction between chlorotetracycline and bovine serum albumin using Eosin y as site marker with the aid of chemometrics. Spectrochim. Acta - Part A Mol. Biomol. Spectrosc..

[21] Birla L, Cristian AM, Hillebrand M (2004). Absorption and steady state fluorescence study of interaction between eosin and bovine serum albumin. Spectrochim. Acta - Part A Mol. Biomol. Spectrosc..

[22] Hallett SA, Ono W, Ono N (2019). Growth plate chondrocytes: Skeletal development, growth and beyond. Int. J. Mol. Sci..

[23] Akiyama H, Chaboissier MC, Martin JF, Schedl A, De Crombrugghe B (2002). The transcription factor Sox9 has essential roles in successive steps of the chondrocyte differentiation pathway and is required for expression of Sox5 and Sox6. Genes Dev..

[24] Yakar S, Werner H, Rosen CJ (2018). Insulin-like growth factors: Actions on the skeleton. J. Mol. Endocrinol..

[25] Farquharson C, Loveridge N (1990). Cell proliferation within the growth plate of long bones assessed by bromodeoxyuridine uptake and its relationship to glucose 6-phosphate dehydrogenase activity. Bone Miner..

[26] Aizawa T, Kokubun S, Tanaka Y (1997). Apoptosis and proliferation of growth plate chondrocytes in rabbits. J. Bone Joint Surg. Br..

[27] Tchetina E, Mwale F, Poole A (2014). Changes in gene expression associated with matrix turnover, chondrocyte proliferation and hypertrophy in the bovine growth plate. Acta Naturae.

