# Supplementary material for: A simple method based on confocal microscopy and thick sections recognizes seven subphases in growth plate chondrocytes
Source: Sci Rep. 2020 Apr 24;10:6935. doi: 10.1038/s41598-020-63978-6 (PMC7181680; doi:10.1038/s41598-020-63978-6)
Supplement: Supplementary file 5 — Supplementary Methods. [file 41598_2020_63978_MOESM5_ESM.docx]

SUPPLEMENTARY METHODS

Title: A simple method based on confocal microscopy and thick sections recognizes seven subphases in growth plate chondrocytes.

Authors: Ángela Fernández-Iglesias, Rocío Fuente, Helena Gil-Peña, Laura Alonso-Duran, María García-Bengoa, Fernando Santos and José M López

Tissue collection

The tibia and femur of each leg were separated at the knee joint immediately after death and were then bisected sagittally with the aid of a stereomicroscope using high quality sharp blades. Next, the two halves of each bone were cut again parallel to the parasagittal plane to obtain two slices of about 2 mm in thickness, a medial one and a lateral one. Two medial slices and two hemispherical-like caps of about 2 mm in thickness were obtained from each bone. Medial slices were used to compare the effectiveness of a series of fixation and staining solutions whereas hemispherical-like caps were used as negative controls to characterize background fluorescence. One of the four hemispherical-like caps in each bone was not fixed and embedded without staining to control fluorescence due to the intrinsic biomolecules of the chondrocytes (native autofluorescence) and the other three were fixed each with one of the three fixatives (see later) and embedded without staining to control fluorescence due to fixation (induced autofluorescence). A total number of forty-eight tibial samples and forty-eight femoral samples were obtained for positive analysis and the same number for background fluorescence controls. Bone slices were placed without delay in the fixative solution to minimize the time lapse between surgical excision of the specimen and fixation. Fixation, staining and dehydration were carried out at 4ºC to retard autolysis and prevent extraction of cytoplasm material during the procedure. Likewise, the osmolarity of the solutions were always adjusted to be isotonic with the tissue (300 mOsm) in order to preserve the normal volume/morphology of the chondrocytes. Samples were individually placed in glass vials 10 ml, 24.5 mm diameter x 41 mm high with clip-on caps (Taab G060). Samples remained in the same glass vial until they were moved into the moulds for polymerization. The procedure does not include demineralization, or conventional sectioning. It consists of a sequence of five steps: (1) fixation, (2) In-block staining, (3) embedding and block formation, (4) ground section preparation, and (5) analysis with confocal microscopy.

Fixation

Three fixative solutions were tested, all containing ruthenium hexaammine trichloride (RHT) and glutaraldehyde and adjusted to 300 mOsm^28,29^ :

F1: Paraformaldehyde 0.5%, glutaraldehyde 1.2 % and ruthenium hexaammine trichloride 0.5% in 0.025 M sodium cacodylate buffer (pH 7.4).

F2: Glutaraldehyde 2.5 % and ruthenium hexaammine trichloride 0.7% in 0.03 M sodium cacodylate buffer (pH 7.4).

F3: Glutaraldehyde 2%, ruthenium hexaammine trichloride 0.5% and calcium chloride 5mM in 0.025 M sodium cacodylate buffer (pH 7.4).

Fixatives were always freshly prepared and cooled to 4°C prior to use. The pH was adjusted to 7.4 with cacodylate buffer, which does not tend to form precipitates in presence of calcium ions (Hayat 1981). Paraformaldehyde was restricted to low concentrations because a 1% unbuffered solution exerts an osmotic pressure of 300 mOsm. It was always dissolved in hot water immediately before use to keep the concentrations of polymers low. Calcium chloride (MgCl2) was included in one fixative because of its effect on phospholipid preservation^30^ and also to test a hypothetical contribution of the divalent calcium cation to aggrecan precipitation and shrinkage prevention. Total bone samples were divided in three equal groups, each containing sixteen tibial samples and sixteen femoral samples, and each group was treated with one of the three fixative solutions. Bone samples were individually placed in tubes containing 5 ml of the fixative solution for 12 hours at 4°C. After this time, the fixative was decanted and replaced by new/fresh fixative solution in which tissues remained 5 additional hours (4ºC). Samples were then washed three times of 30 min each with the post fix buffer consisting of sodium cacodylate 0.1 M, MgCl2 3mM and sucrose 2.5% (osmolarity 300 mOsmols, 4ºC). The quality of preservation of the hypertrophic chondrocytes in each fixative solution was subsequently evaluated under the confocal microscope, as detailed later.

In-block staining

Epoxy-resin was used as embedding medium. Tissue staining was performed prior to embedding. Eosin Y was assessed as a potential fluorescent probe for in-block staining. This xanthene dye obtained by halogenization of fluorescein has high‐fluorescence emission and selectively marks cell constituents of protein nature because of the electrostatic interactions between its carboxylic and phenolic groups to arginine, histidine, lysine, and tryptophan residues of proteins. Since both the spectral behavior of eosin and its fluorescence quantum yield slightly vary in different solvent media^31^, several eosin solutions and incubation times were tested. Bone samples were incubated with two eosin concentrations (0.5 and 1%) at two different pH (4.8 and 5.5) and during two staining times (2 and 4 hours), so a total of eight possible combinations were tested. In order to not increase excessively number of staining solutions, eosin was always diluted in acetate buffered ethanol 60% (based on previous experience in our lab) and both staining and post-staining washes were always carried out at 4°C. After staining, bone samples were washed two times of 15 min each with ethanol 80% followed by two changes of 15 mins each in ethanol 90%, two changes of 30 mins each in ethanol 100% and two changes of 30 mins each in acetone. With the aim of evaluate the effect of the fixative on downstream staining, the samples were subdivided in a total of twenty-four groups (n=4, two tibias and two femurs) and each was treated with one of the combinations of three fixative solutions and the eight staining conditions (Table 1). The quality of the fluorescent signal was subsequently evaluated in each group under the confocal microscope, as detailed later.

Embedding

Bone samples were embedded in epoxy resin. A soft Durcupan ACM mixture (Sigma) was chosen based on previous experience in our laboratory. Infiltration was performed in a mixture consisting of 12.2 g epoxy monomers (A/M), 9.5 g hardener (B) and 0.3 g plasticizer (D) that was prepared at least 15 minutes before use. It was applied to the bone samples by first mixing with equal parts of acetone. Bone samples remained in resin/acetone for 2 hours at room temperature. Then, the mixture was removed from each glass tube and replaced by pure resin mixture. Tubes were then incubated at 40°C for 5 hours. Afterwards, the resin was wiped and replaced by a new mixture with the same components as the primary plus 0.4 g of accelerator (C). This mixture was prepared 15 minutes before use and warmed to 40°C. After the samples had remained in this last mixture for 5 hours at 40 ºC, they were individually placed in silicone rubber molds with round cavities of 14 mm diameter x 3 mm deep (Taab E070) filled with freshly prepared resin. Each bone sample was carefully positioned so that it laid perfectly flat against the bottom of the mold to ensure that the block was properly oriented. Next, samples were put in an oven at 50°C and allowed to polymerize for 48 h. After this time, the molds were taken out of the oven and allowed to cool at room temperature before demolding.

Grinding for thick sections

The embedded bone samples appeared as a 3 mm thick disc with one slightly concave face corresponding to the upper side of the mold and a flat one corresponding to the bottom. They were mounted on a specimen holder (Struers AccuStop) with the concave face oriented upwards and grinding was performed by using a commercial grinding machine (LaboPol-1, Struers A/S, Ballerup, Denmark) to a thickness of 0.5 mm. Fine sandpaper with a constant grit size of P800 was used (Buehler GmbH, Düsseldorf, Germany). Once grinded, the sample was glued by the no-grinded face to a histological slide with cyanoacrylate. To eliminate the effect of grinding contamination, the slide was mounted on an automated microtome with tungsten carbide blades (Technovit® Histoblade Ref 66045730) and 1 μm thick sections were made to carefully trim the tissue down to a level where the tissue had no sign of grinding. Finally, Permount mounting media (Permount) was applied and the slides coverslipped and stored in a dark box until observation.

Immunohistochemistry

Fluorescent immunodetection of Sox9, Col2a1, Col10a1, IGF-1, NKCC1, AQP1 was performed in 5 μm thick sections of methyl-methacrylate embedded left tibias. Antigen retrieval was performed with citrate buffer pH 8, 35 min at 60°C for Sox9, proteinase K 1mg/ml 15 min at 37ºC for Col2a1, proteinase K 1mg/ml 10 min at 37ºC for ColX, proteinase K 1mg/ml 16 min at 37°C for Igf-1, hyaluronidase 2mg/ml 30 min 37°C for NKCC1, citrate buffer pH 8 30 min at 60°C for Aqp1. TNB blocking buffer (75 min, RT) made from TSA blocking reagent (Perkin Elmer, MA, USA) was used. The primary antibodies used were anti-Sox9 (#PA5-81966, Invitrogen, California, USA) at 1:50 dilution, anti-Col2a1 (#MA5-12789, Invitrogen, California, USA) at 1:20 dilution, anti-ColX (A6889, Abclonal, MA 01801, USA) at 1:20 dilution, anti-IGF-I (#PA5-27207, Invitrogen, California, USA) at 1:200 dilution, anti-Aqp1 (ab15080, Abcam, Cambridge, UK) at 1:100 dilution and anti-NKCC1 (ab59791, Abcam, Cambridge, UK) at 1:100 dilution. Goat anti-mouse Alexa 594 (A-21155, Invitrogen, California, USA), goat anti-rabbit Alexa 594 (#A-11012, Invitrogen, California, USA) and goat anti-rabbit Alexa 488 (A11034, Invitrogen, California, USA) were used as secondary antibodies and sections were finally mounted with Vectashield Mounting Medium with DAPI (Vector Laboratories, Burlingame, California, USA). Sections were examined and pictures taken with a confocal microscope Leica TCS SP8 (Leica Microsystems, Germany) with the 20x magnification objective. Image J (National Institutes of Health, Bethesda, Maryland, USA) was used to measure the fluorescence intensity on whole chondrocyte columns.

References in supplementary information

28. Hunziker, E. B., Lippuner, K. & Shintani, N. How best to preserve and reveal the structural intricacies of cartilaginous tissue. Matrix Biol. 39, 33-33 43 (2014).

29. Hunziker, E. B., Herrmann, W. & Schenk, R. K. Ruthenium hexamine trichloride (RHT)-mediated interaction between plasmalemmal components and pericellular matrix proteoglycans is responsible for the preservation of chondrocytic plasma membranes in situ during cartilage fixation. J. Histochem. Cytochem. 31, 717–727 (1983).

30. Baker, J. R. The Structure and Chemical Composition of the Golgi element. Quart. J. Micr. Sci 85, 1–71 (1944).

31. Fleming, G. R., Knight, A. W. E., Morris, J. M., Morrison, R. J. S. & Robinson, G. W. Picosecond fluorescence studies of xanthene dyes. J. Am. Chem. Soc. 99, 4306–4311 (1977).

Legends for supplementary videos

Supplementary Videos 1.

Representative video of a three-dimensional-reconstruction of Z-series confocal images of a thick bone section imaged with the confocal microscope. Video was made via the “Movie Maker” function with the increase in display time in association with the depth of the optical section. Travel through xy-projections of a confocal z-stack.

Supplementary Video 2.

Representative video of a three-dimensional-reconstruction of Z-series confocal images of a thick bone section imaged with the confocal microscope. Video was made via the “Movie Maker” function with the increase in display time in association with the depth of the optical section. Travel through xz-projections of a confocal z-stack.

Supplementary Video 3.

Representative video of a 3D reconstruction of chondrocytes at proliferative and early hypertrophic zones tracked in successive confocal planes obtained with IMARIS software image software.

Supplementary Video 4.

Representative video of a 3D reconstruction of chondrocytes at the hypertrophy zone tracked in successive confocal planes obtained with IMARIS software image software.
